# Adipose tissue for reconstruction and wound healing: applications, challenges, and future directions

**DOI:** 10.3389/fcell.2026.1886380

**Published:** 2026-07-23

**Authors:** Karolina Kondej, Paulina Słonimska, Aneta Skoniecka, Agata Tymińska, Agnes S. Klar, Michał Pikuła

**Affiliations:** 1 Department of Surgery and Urology for Children and Adolescents, Medical University of Gdansk, Gdańsk, Poland; 2 Department of Clinical Anatomy, Medical University of Gdansk, Gdańsk, Poland; 3 Laboratory of Tissue Engineering and Regenerative Medicine, Division of Embryology, Medical University of Gdansk, Gdańsk, Poland; 4 Tissue Biology Research Unit, University Children’s Hospital Zurich, Zurich, Switzerland; 5 Faculty of Medicine, University of Zurich, Zurich, Switzerland; 6 Department of Biochemistry, Gdansk University of Physical Education and Sport, Gdańsk, Poland

**Keywords:** adipose-derived stem cells, fat/adipose tissue, regenerative medicine, stem cells, stromal vascular fraction

## Abstract

Adipose tissue has emerged as an important therapeutic resource in regenerative medicine due to its accessibility and its rich content of regenerative cells and bioactive factors. Adipose-derived products, including lipoaspirate, stromal vascular fraction (SVF), and adipose-derived mesenchymal stromal/stem cells (AD-MSCs), are increasingly used in clinical practice for tissue reconstruction, wound healing, and the treatment of complex tissue defects. In this review, we summarize current clinical applications of adipose tissue–based therapies, focusing on their role in tissue regeneration and wound management. We briefly discuss adipose tissue harvesting techniques and the biological characteristics of AD-MSCs relevant to their therapeutic use. Attention is given to clinical evidence regarding efficacy, safety, and translational challenges associated with SVF- and AD-MSC-based interventions. We highlight current limitations and future directions for the integration of adipose tissue–derived therapies into regenerative and reconstructive medicine.

## Introduction

Adipose tissue was traditionally perceived as a passive energy-storage depot. However, it is now recognized as an active endocrine organ that contributes to the regulation of metabolism and immune-system processes. Adipocytes’ cytoplasm is almost entirely filled with lipid droplets, primarily in the form of triglycerides. This tissue is well vascularized and characterized by high plasticity, with its volume varying depending on diet, physical activity, and environmental conditions. Adipose tissue exhibits endocrine activity: it secretes adipokines that influence energy management and metabolism throughout the body (Booth et al., 2016).

Additionally, adipose tissue is a readily available, biocompatible, and autologous material, minimizing the risk of rejection and immunological complications (Peng et al., 2020).

In recent years, there has been a growing interest in utilizing cells derived from adipose tissue as a material for skin reconstruction. Driven by significant needs in wound management and tissue repair. Chronic and non-healing wounds, including diabetic ulcers, pressure sores, and extensive burn injuries, represent a major clinical challenge. These wounds often persist despite standard surgical and conservative therapies, resulting in prolonged morbidity, an increased risk of infection, and substantial healthcare costs. Traditional treatments, including skin grafts and flap reconstructions, often provide suboptimal outcomes in terms of functional recovery and cosmetic appearance, and are associated with limitations such as donor site morbidity and graft failure. Adipose-derived mesenchymal stromal/stem cells (AD-MSCs) have thus attracted substantial interest because they can be readily obtained from abundant autologous adipose tissue via minimally invasive procedures, yielding a high number of stem/progenitor cells for therapeutic use. The number of clinical studies investigating AD-MSCs has increased substantially over the last decade. This growing interest reflects both their regenerative potential, including the promotion of angiogenesis, immunomodulation, and re-epithelialization, and the increasing demand for innovative therapies in plastic and reconstructive surgery.

The clinical need for such therapies is particularly evident in patients with chronic refractory wounds that fail to respond to conventional treatment and significantly reduce quality of life. Similarly, soft tissue defects resulting from trauma, tumor resection, or burns remain challenging to manage, highlighting the need for strategies that enhance tissue regeneration while minimizing scar formation (Gentile and Garcovich, 2021; Li et al., 2024).

## Lipofilling and difficult-to-heal wounds

Adipose tissue is a valuable source of cells with regenerative properties. These cells can be obtained from the stromal vascular fraction (SVF), a heterogeneous mixture isolated from adipose tissue via enzymatic digestion. Stromal cells may be isolated from adipose tissue using either enzymatic or mechanical methods; however, SVF is typically prepared using an enzymatic approach, in which enzymes break down the dense extracellular matrix of adipose tissue. The stromal cell fraction is subsequently isolated by centrifugation (Copcu and Oztan, 2021).

The combination of adipose tissue and its derived stromal/stem cells offer a synergistic strategy for regenerative therapies. Such cell-enriched fat grafts are increasingly used in the treatment of chronic wounds, soft tissue defects, and scar remodeling. Collectively, these approaches leverage both the structural properties of adipose tissue and the biological activity of mesenchymal stromal/stem cells, leading to improved healing outcomes in patients with difficult-to-treat wounds (Zeng et al., 2022). Due to the limitations of traditional methods, cell-assisted lipotransfer (CAL) is becoming increasingly popular (Zeng et al., 2022).

**FIGURE 1:**
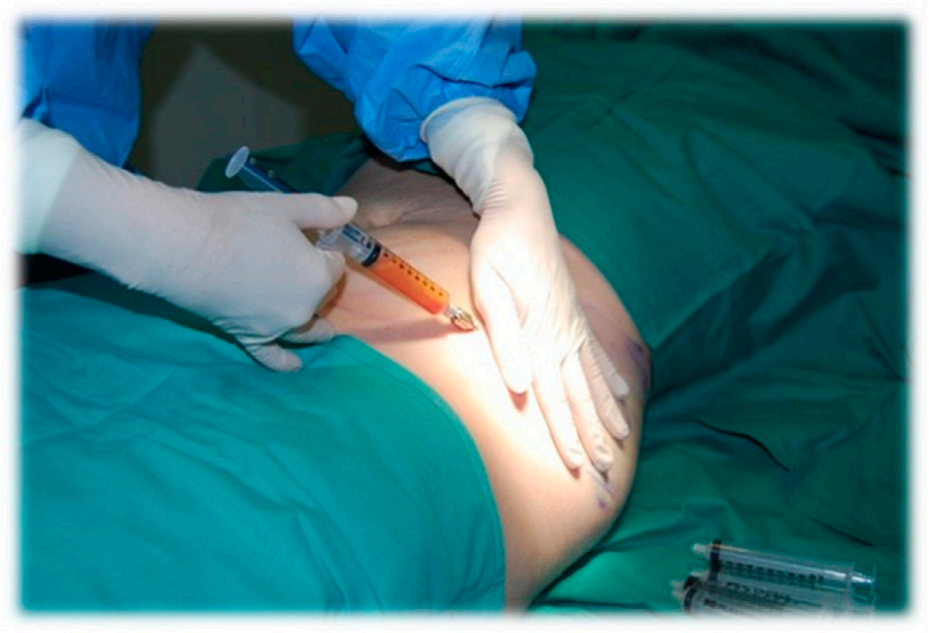
Abdominal regions are commonly used as a donor site for adipose tissue harvesting prior to autologous fat grafting or isolation of adipose-derived stem/stromal cells (AD-MSCs).

Adipose tissue obtained by liposuction (Figure 1) requires only local anesthesia, which is less traumatic for patients while allowing many stem cells to be harvested (Malekzadeh et al., 2023).

**FIGURE 2:**
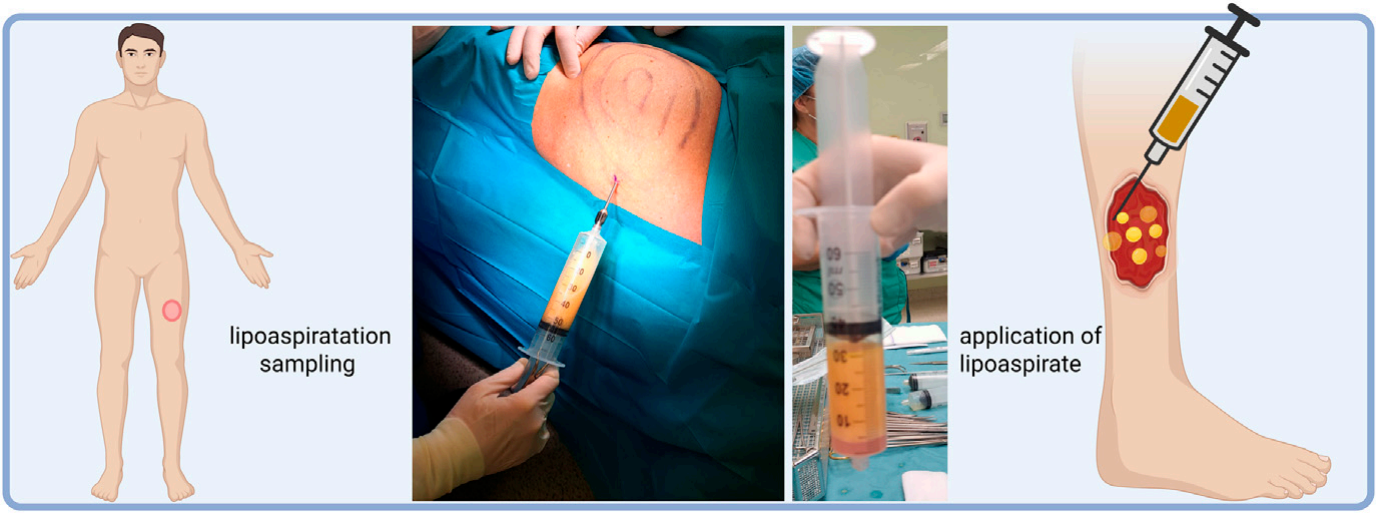
Schematic representation of autologous fat grafting and adipose-derived regenerative therapy. The procedure includes adipose tissue harvesting, lipoaspirate processing, and their administration to damaged tissues. The regenerative effects are mediated through tissue integration, angiogenesis, immunomodulation, and the secretion of bioactive factors (The drawing was created with BioRender.com).

Studies of autologous fat grafting (Figure 2) have shown accelerated healing in chronically scarred tissue, chronic wounds, and radiation-induced soft tissue injuries (Eschborn et al., 2023; Kenny et al., 2021). In the treatment of burns, this therapy promotes wound healing, reduces the typical healing time, and minimizes the occurrence of hypertrophic scarring (Malekzadeh et al., 2023).

Transferred adipose tissue may support the lipid layer, which contributes to defense against skin infections^8,^ including through the production of antimicrobial peptides (Horsley, 2022).

The use of autologous lipotransfer after surgical debridement is becoming increasingly popular in the treatment of burns, scars, and chronic wounds, including diabetic foot ulcers and lower limb ulcers. This growing interest is driven by improved clinical outcomes, such as reduced wound size and faster healing (Stasch et al., 2015). Long-term results have confirmed the effectiveness of autologous fat transfer in the treatment of hypertrophic scarring and burn scars, as well as chronic wounds (Eschborn et al., 2023).

**FIGURE 3:**
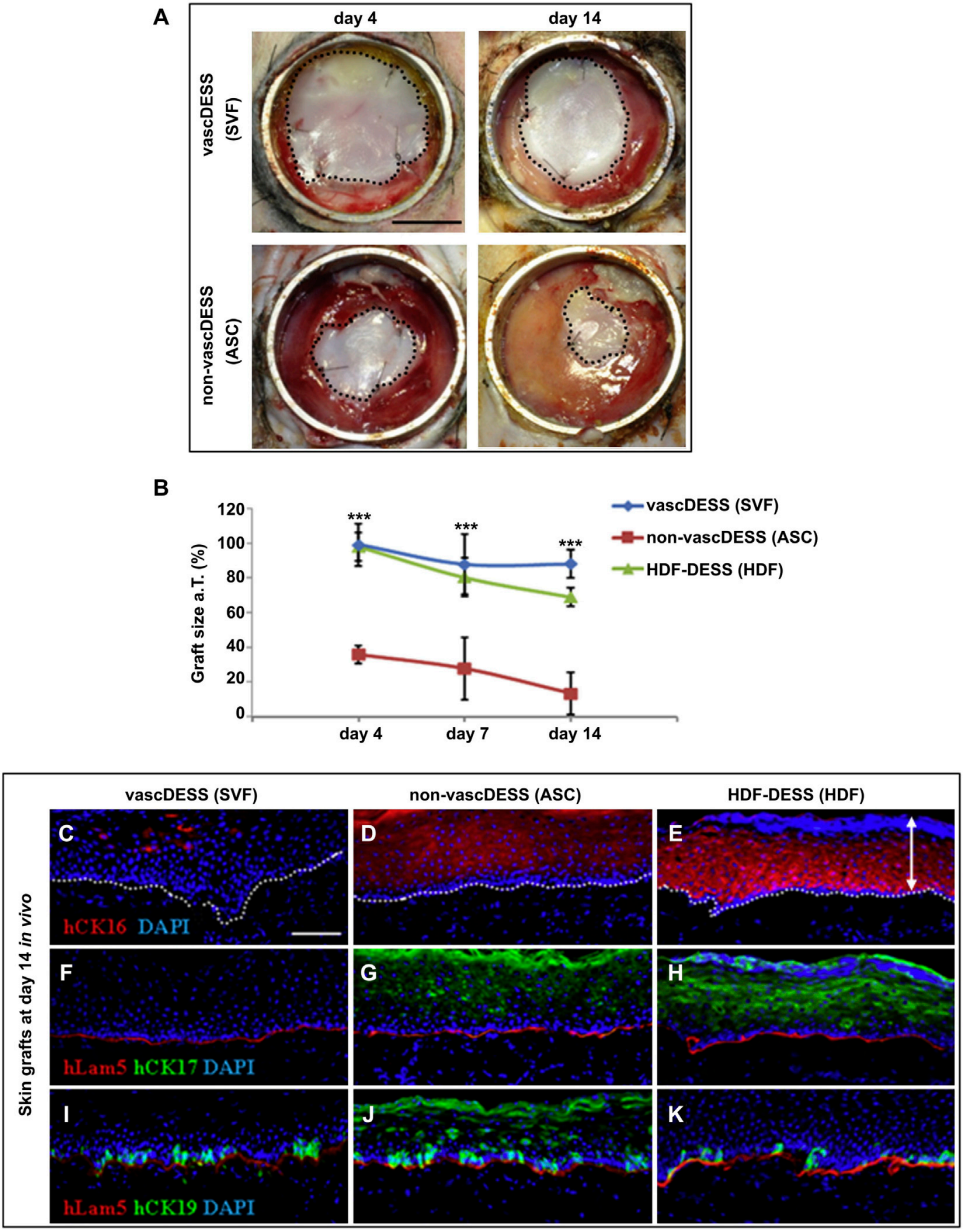
A skin model equipped with a vascular network exhibits reduced tissue contraction and accelerated regeneration compared with avascular control groups. **(A)** Macroscopic view of the skin constructs on days 4 and 14 post‐transplantation. Black dashed lines delineate the areas of individual skin grafts used for planimetric analysis in **(B)**. **(B)** Comparison of graft areas between the vascularized (SVF) and control groups. **(C–K)** Immunofluorescence staining of wound healing and epidermal differentiation markers. DAPI stains nuclei blue. White dashed lines indicate the dermo‐epidermal junction **(C–K)**, and the white arrow indicates epidermal thickness (E). Abbreviations: CK, cytokeratin; HDF, human dermal fibroblasts; Lam5, laminin 5. Scale bars = 1 cm **(A)** and 100 μm **(C–K)**. Overall, tissue morphology and immunofluorescence analysis indicate that vascularization promotes faster wound closure, improved epidermal organization, and earlier restoration of tissue homeostasis. Reproduced from Klar, A. S., Güven, S., Biedermann, T., Luginbühl, J., Böttcher‐ Haberzeth, S., Meuli‐Simmen, C., …and Reichmann, E. (2014). Tissue‐engineered dermo‐epidermal skin grafts prevascularized with adipose‐ derived cells. Biomaterials, 35. (Zocchi et al., 2022), 5065-5078 (Klar et al., 2017).

While clinical studies demonstrate the therapeutic benefits of adipose tissue grafting, experimental models provide insight into the underlying mechanisms of SVF-mediated regeneration. Studies by Klar *et al.* have demonstrated that the stromal vascular fraction is an effective regenerative tool that combines stem cells with supporting cell populations. The pro-regenerative potential of SVF is primarily attributed to its paracrine activity, promotion of angiogenesis, immunomodulatory effects, and limited differentiation capacity. *In vivo* experiments conducted in immunodeficient mice involved implantation of biomaterials alone or enriched with human-derived SVF. Analysis of adipogenesis, angiogenesis, and inflammatory response revealed that SVF significantly enhanced adipose tissue formation within the implants. Importantly, SVF cells not only contributed through differentiation but also recruited host cells. Furthermore, SVF modulated the inflammatory response and strongly stimulated angiogenesis, which is crucial for graft survival (Figure 3).

Despite these promising findings, the translation of SVF-based therapies into clinical practice remains challenging, largely due to the lack of standardization in SVF isolation procedures. Variability in the isolation of SVF using enzymatic and mechanical methods is one of the main challenges in clinical trials involving adipose tissue-derived cells. The quality and composition of the obtained product are influenced by the isolation method, the technical parameters of the procedure, and the biological characteristics of the donor. The enzymatic method, which most commonly uses collagenase to degrade the extracellular matrix, generally enables higher cell yields and greater isolation efficiency; however, the results may vary depending on the type of enzyme used, its concentration, incubation time, and processing temperature. Mechanical methods, based on filtration, fragmentation, or centrifugation of adipose tissue, are simpler and often more easily implemented in clinical settings, but may result in greater variability in cellular composition and lower cell yields (Winnier et al., 2019). Centrifugation parameters, such as centrifugal force, processing time, number of cycles, and temperature, also contribute significantly to variability. Inappropriately selected conditions may affect cell viability, integrity, and the overall quality of the final product. Furthermore, donor-related factors, including age, sex, body mass index (BMI), health status, comorbidities, lifestyle, and the anatomical site of adipose tissue harvesting, play an important role. These factors can influence both the number of cells present in the SVF and their regenerative potential (Sforza et al., 2025).

To ensure reproducible results in clinical studies, additional quality control and standardization measures are required. Tissue collection procedures should be standardized by defining adipose tissue harvesting sites and establishing minimum sample volume requirements. Detailed isolation protocols should also be implemented, including precisely defined enzymatic or mechanical conditions and standardized centrifugation parameters. Regular calibration of laboratory equipment and monitoring of all processing steps are essential. The final product should undergo routine quality assessment, including evaluation of cell count, viability, cellular composition, and functional potential. Furthermore, the implementation of standard operating procedures (SOPs) across research centers, personnel training, and interlaboratory quality control programs is recommended to facilitate comparison of results between laboratories. Standardization of isolation methods, technical parameters, and reporting practices is crucial for obtaining reliable, comparable, and reproducible results, thereby supporting the further development of SVF-based therapies in clinical practice (Sforza et al., 2025).

## Therapeutic properties of lipoaspirate

**FIGURE 4:**
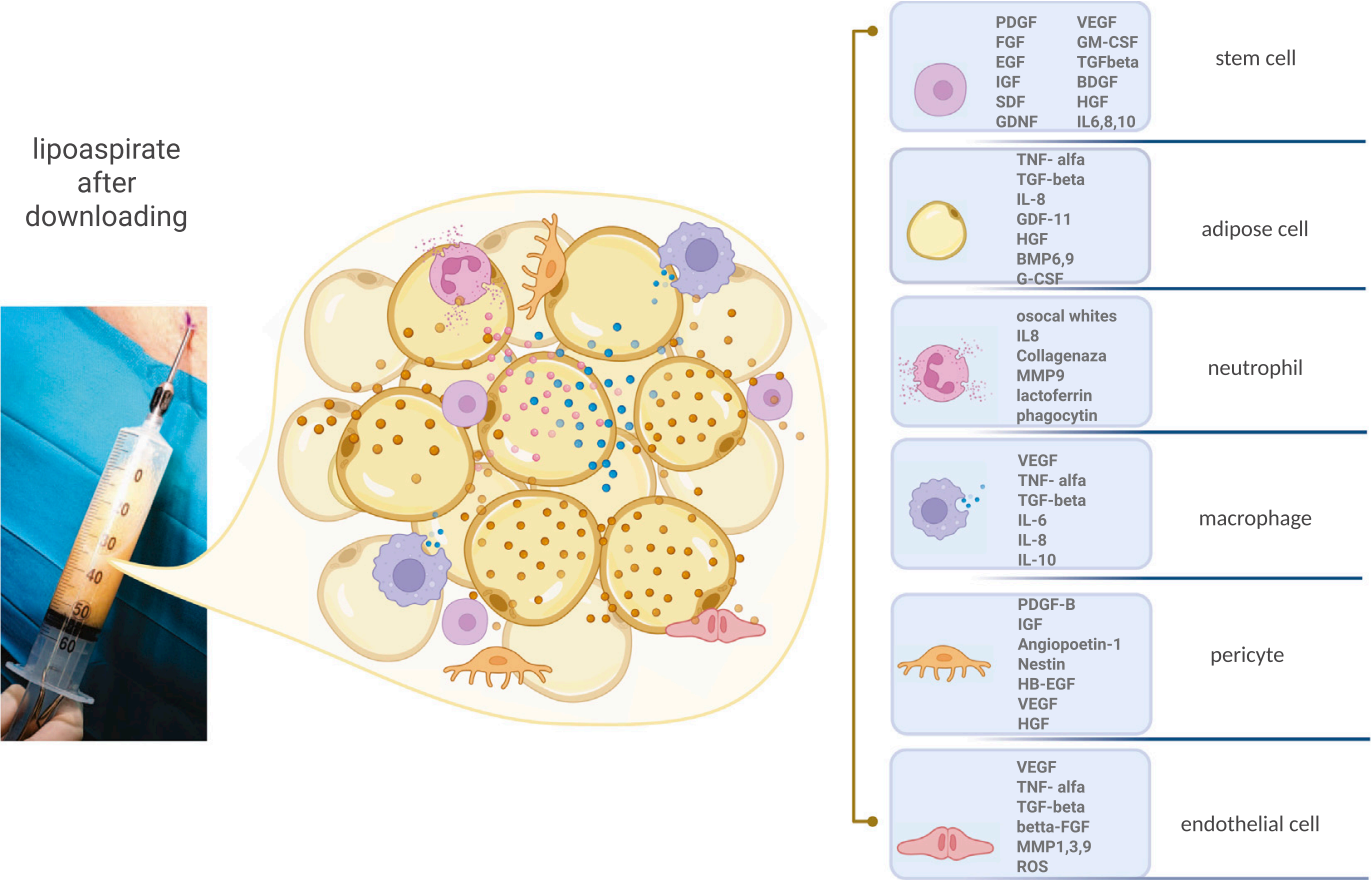
The basic mechanism by which autologous lipoaspirate promotes wound healing. Lipoaspirate is a heterogenous group of cells secreting different cytokines and growth factors (The drawing was created with BioRender.com).

As lipoaspirate contains a wide variety of cellular substances—such as the pro-inflammatory cytokines IL-6, IL-8, and IL-11; TNF-α; the anti-inflammatory prostaglandin E2; and growth factors including VEGF, HGF, FGF, G-CSF, and GM-CSF (Figure 4). It can promote regenerative processes at the target site (Doornaert et al., 2019).

Under hypoxic conditions, AD-MSCs increase their paracrine secretion of cytokines, thereby promoting angiogenesis, which is essential for supplying oxygen and nutrients to both transplanted cells and the wound microenvironment. Additionally, this may support revascularization, tissue remodeling, and multilineage differentiation (Bellini et al., 2017; Abu-Ghname et al., 2019; Khouri and Khouri, 2017).

In 2014, Ghosh described AD-MSC-assisted lipotransfer, known as cell-assisted lipotransfer (CAL), as an approach that may enhance collagen synthesis, angiogenesis, tissue remodeling, and regeneration at the recipient site^17^. AD-MSCs can modulate the local tissue microenvironment by becoming activated during the regenerative process and establishing a regenerative niche. Through metabolic and chemotactic signaling, they facilitate communication between transplanted cells and recipient tissues, thereby supporting tissue repair and integration (Zocchi et al., 2022).

## Combining PRP, SVF, and CAL with lipoaspirate

Experiments combining several cell fractions of SVF, PRP, and CAL (AD-MSC) with lipoaspirate to enhance therapeutic properties are receiving increasing attention (Liao et al., 2014).

Supplementation with SVF fractions can prolong lipotransfer survival, mainly through angiogenesis. Improved integration of the implanted fat into recipient tissues, along with paracrine secretion of factors that induce collagen and elastin production and reduce fibrosis, has also been reported (Borrelli et al., 2019). In other studies, the addition of AD-MSCs improved the volume retention of implanted fat grafts, increasing it by approximately 40% compared with grafts without AD-MSCs (Kølle et al., 2020). In Kamakura’s study of lipotransfer with AD-MSCs in 20 patients undergoing breast augmentation, breast volume improved and remained stable up to 9 months post-procedure (Kamakura and Ito, 2011). Many reports describe AD-MSC CD34+/CD146+ subpopulations as promoting angiogenesis through VEGF expression, and CD34+/CD74+ subpopulations as possessing anti-fibrotic properties associated with reduced TGF-β1 expression (Borrelli et al., 2020).

## PRP/ECM/SVF/PRF

Platelet rich plasma (PRP) is widely used in plastic surgery as a graft support material (Caruana et al., 2019). High concentrations of platelets and growth factors can be combined with SVF fraction when a high concentration of regenerative components in a small volume is required. PRP can provide a reservoir of pro-angiogenic factors that stimulate vasculogenesis and prevent apoptotic effects. PRP administration can increase the survival of transferred fat by decreasing the effects of the ischemic phase of transplantation (Smith et al., 2017). As Sonker cites the antimicrobial and immunomodulatory properties, PRP can facilitate healing and reduce wound infection (Sonker et al., 2015).

PRP concentrate promotes healing and shows up to 8-fold increase in platelet concentration compared to whole blood, 5-fold increase in PDGF-BB, 3-fold TGFβ increase, and 6-fold vascular endothelial growth factor concentration (Eppley et al., 2004).

Zocchi *et al.* proposed to adapt procedures for the creation of bioactive composite mixtures (BACM) depending on the clinical needs and type of recipient tissue as well as the anatomical structure of the recipient. The BACM can contain cellular components:

- an adipogenic SVF so-called tissue SVF (TSVF) with ECM, or
- cellular CSVF has a progenitor cell pool or
- concentrated SVF, or
- a pure AD-MSC precursor cell fraction.

In addition, it proposed to use platelet-rich fibrin (PRF) and platelet (PL) as growth factor-secreting components. Such components must have adequate amino acids, vitamins, and glutathione (GSH) to ensure cell growth and facilitate implantation of hyaluronic acid, and ECM. Cellular and blood components are key in bioactive complex therapies (BACT). The same author, in a 2017, highlights 3D macro- and microclusters obtained via mechanical isolation, which yield the best-functioning grafts due to their structural integrity, support for remodeling, protection of new niches at the recipient site, and low transplantation costs (Zocchi and Zocchi, 2017). Growth factors released by platelet activation from blood components promote chemotaxis, proliferation, differentiation, and angiogenesis in a highly controlled manner by releasing fibronectin, nectin *in vitro,* and sphingosine 1-phosphate (Kobayashi et al., 2016; Xiao et al., 2024).

## AD-MSCs in wound healing

The wound healing process can be divided into four main overlapping stages: homeostasis, inflammation, proliferation (granulation tissue formation and re-epithelialization), and remodeling or scar maturation. Virtually all types of skin cells are involved in this process (Richardson et al., 2013). Growth factor-mediated cellular interactions between fibroblasts, myofibroblasts, keratinocytes, smooth muscle cells, endothelial cells, and immune cells play diverse roles in wound healing and regeneration (Singh et al., 2017).

**FIGURE 5:**
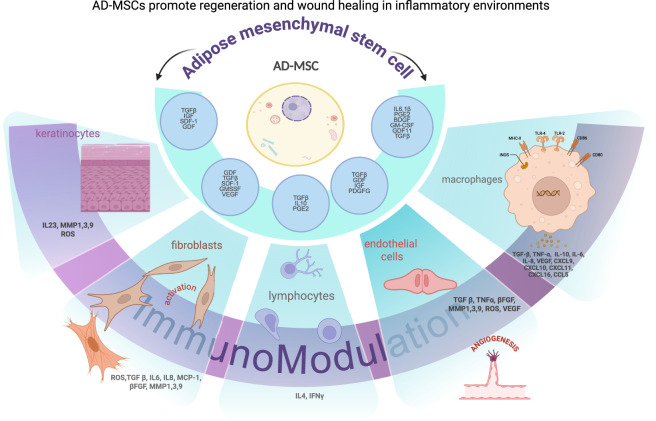
Growth factors, cytokines, and extracellular vesicle-associated molecules secreted by AD-MSCs that participate in wound healing and scar remodelling. These mediators promote angiogenesis, modulate inflammatory responses, regulate fibroblast activity, and support extracellular matrix remodelling (The drawing was created with BioRender.com).

During the proliferation and remodeling stages, AD-MSCs secrete biological factors such as PDGF, IGF, VEGF, HGF, and TGF-β, which promote fibroblast proliferation and migration, angiogenesis, and the synthesis of collagen and other extracellular matrix proteins that have beneficial effects on the skin (Zeng et al., 2022; Pankajakshan and Agrawal, 2014), as shown in Figure 5.

AD-MSCs can be used to promote neovascularization following radiotherapy, which can cause severe local skin ischemia (Zeng et al., 2022). Stem cell-based therapy after radiotherapy can reduce radiation-related skin complications and prevent or treat radiation-induced skin damage (Zeng et al., 2022; Zeinalzadeh et al., 2025). Moreover, AD-MSCs can reduce extracellular matrix degradation by secreting tissue metalloproteinase inhibitors (Zeng et al., 2022). Extracellular matrix proteins (such as PDGF and TGF-β) can protect growth factors and cytokines produced by activated platelets and macrophages from degradation (Zeng et al., 2022). *In vitro* studies have also confirmed the role of AD-MSCs in promoting re-epithelialization by regulating keratinocyte proliferation and migration (Zeng et al., 2022). AD-MSCs can promote wound healing by reducing inflammation, inducing angiogenesis, promoting fibroblast and keratinocyte growth, and supporting extracellular matrix production (Zeng et al., 2022).

Injection of AD-MSCs significantly reduces levels of type I collagen, type III collagen, TGF-β1, and smooth muscle α-actin in hypertrophic scar (which are usually characterized by an abnormal extracellular matrix (ECM), excessive collagen deposition, and an abnormal arrangement)^8^. AD-MSCs exhibit anti-fibrotic effects by downregulating the TGF-β1/Smad signaling pathway, and decreased decorin (DCN) expression, which effectively inhibit fibroblast contraction and collagen synthesis (Li et al., 2024). The use of AD-MSCs may indeed regulate the early stages of scar formation and remodeling, and the improvement in scar formation is associated with TGF-β inhibition (Li et al., 2024; Laloze et al., 2021).

## AD-MSCs–immunomodulatory properties

Beyond their regenerative potential, AD-MSCs possess potent immunomodulatory properties and low immunogenicity, characterized by low HLA-I expression and the absence of HLA-II. These features contribute to their broad therapeutic applicability. AD-MSCs promote wound healing by modulating immune responses, including the induction of regulatory T cells and polarization of macrophages toward an anti-inflammatory M2 phenotype. They also stimulate fibroblast proliferation, support angiogenesis, reduce fibrosis, and limit scar formation (Malekzadeh et al., 2023).

The use of stem cells offers great potential in improving wound healing outcomes and advances in the field of burn treatment (Malekzadeh et al., 2023). AD-MSCs promote wound healing by modulating the immune response, secreting paracrine factors and promoting angiogenesis (Gruca et al., 2022). In physiological injury, endogenous stem cells are activated, migrate to the site of injury, and the wound healing process is enhanced through paracrine interactions with local and immune cells (Malekzadeh et al., 2023). Secreted cytokines and growth factors modulate the wound microenvironment, reduce inflammation, and promote angiogenesis, cell proliferation, and extracellular matrix deposition (Malekzadeh et al., 2023).

AD-MSCs can migrate into damaged skin to regenerate aging cells and repopulate the tissue (Gruca et al., 2022). Through the secretion of cytokines such as TGF-β, VEGF, GM-CSF, and HGF, AD-MSCs can recruit and stimulate endogenous stem cells to participate in the wound healing process (Gruca et al., 2022).

The table below provides a summary of the methods described above (Table 1).

**TABLE 1:** Summary of the methods.

Therapy	Description	Mechanism in wound healing	Advantages	Disadvantages/Limitations
Lipoaspirate	Minimally processed fat tissue obtained via liposuction, containing adipocytes, stromal cells, and vascular components	Provides a natural scaffold, releases paracrine growth factors, improves angiogenesis and tissue regeneration	Autologous, rich cellular microenvironment, structural support for chronic wounds, relatively simple processing	Variable cell composition, limited standardization, required liposuction, bulky tissue may not integrate well in all wounds
SVF (Stromal Vascular Fraction)	Heterogeneous cell population isolated from adipose tissue (including AD-MSCs, endothelial cells, pericytes, immune cells)	Promotes angiogenesis, modulates inflammation, enhances fibroblast activity and re-epithelialization via paracrine signaling	High regenerative potential, strong pro-angiogenic and immunomodulatory effects, autologous	Requires enzymatic or mechanical processing, regulatory restrictions in many countries, and lack of standardization
PRP (Platelet-Rich Plasma)	Concentrated platelets derived from autologous blood	Releases platelet-derived growth factors (PDGF, VEGF, TGF-β) to stimulate cell migration, angiogenesis, and proliferation	Easy to obtain, autologous, relatively inexpensive, well-documented safety profile	Short-lived effect, variability between patients, limited efficacy in ischemic or infected chronic wounds
CAL (cell-assisted lipotransfer)	Adipose-derived cells expanded *in vitro*, enriched in regenerative cell populations	Enhances wound healing through increased cell numbers, angiogenesis, and extracellular matrix remodeling	Higher cell concentration, potentially stronger regenerative effects	Requires cell culture facilities, higher cost, regulatory and ethical constraints, risk of contamination
AD-MSCs (Adipose-derived mesenchymal stromal/stem cells)	Isolated and characterized mesenchymal stem cells from adipose tissue	Potent paracrine signaling, immunomodulation, stimulation of angiogenesis, fibroblast activity, and keratinocyte migration	High regenerative capacity, anti-inflammatory effects, promising results in chronic and complex wounds	Requires advanced processing and culture, regulatory barriers, high cost, limited long-term clinical data
Adipose-derived MSC Exosomes	Cell-free extracellular vesicles are secreted by adipose-derived mesenchymal stem cells (AD-MSCs), containing proteins, lipids, mRNA, and microRNA with regenerative properties	Promote angiogenesis through VEGF-related signaling, enhance fibroblast proliferation and migration, modulate inflammation by shifting macrophages toward an anti-inflammatory phenotype, stimulate collagen deposition, and support tissue remodeling	Cell-free approaches with lower immunogenicity and tumorigenic risk than cell therapies; easier storage and handling; reduced regulatory concerns compared with live-cell products	Lack of standardized isolation and characterization methods; variability in exosome content; limited large-scale clinical evidence; challenges in dose optimization and manufacturing consistency
Acellular Adipose Matrix (AAM)	Decellularized adipose tissue-derived extracellular matrix scaffold retaining structural proteins and bioactive components while removing cellular material	Provides a three-dimensional scaffold for cell migration and tissue regeneration, supports neovascularization, enhances cell attachment and proliferation, and promotes constructive tissue remodeling	Biocompatible and minimally immunogenic; provides structural support; can be combined with stem cells or growth factors; avoids risks associated with live-cell transplantation	Variable degradation rates; limited intrinsic regenerative activity without additional biologic stimulation; lack of standardized preparation protocols; relatively limited long-term clinical data

## Comparison of regenerative therapies using adipose tissue with other tissues

Stem cells have been isolated not only from adipose tissue but also from tendons, periodontal ligaments, synovial membranes, trabecular bone, bone marrow, the nervous system, skin, periosteum, and muscle. Adult tissues contain specific stem cell niches that provide replacement cells during normal cell turnover and tissue regeneration following injury. The epidermis, hair follicles, hematopoietic tissue, and the gastrointestinal tract are well-known examples of tissues maintained by stem cell niches (Orbay et al., 2012).

One of the main advantages of AD-MSCs is the ease of biological material collection. Adipose tissue can be obtained through liposuction, a relatively minimally invasive procedure that yields many cells. In contrast, the isolation of BM-MSCs requires bone marrow aspiration, most commonly from the iliac crest, which is associated with greater invasiveness, post-procedural discomfort, and a limited amount of harvested material. Furthermore, the number of MSCs present in bone marrow decreases with donor age, whereas adipose tissue remains a rich source of progenitor cells even in elderly individuals (Fraser et al., 2006). Dermal fibroblasts can also be obtained relatively easily through skin biopsy (Iannello et al., 2023); however, their regenerative potential is more limited than that of mesenchymal stem cells. In terms of proliferation and *in vitro* expansion capacity, AD-MSCs generally exhibit higher proliferative activity than BM-MSCs. Consequently, enough cells for therapeutic applications can be obtained within a shorter period. Fibroblasts also proliferate efficiently in culture; however, their differentiation potential is considerably lower because they are more specialized and functionally committed cells. An important characteristic of AD-MSCs is their multilineage differentiation potential (Mazini et al., 2019; Czerwiec et al., 2023). Immunomodulation represents another important aspect of AD-MSCs biology. These cells can regulate immune responses through interactions with T lymphocytes, B lymphocytes, macrophages, and dendritic cells. Consequently, they have applications not only in tissue regeneration but also in the treatment of inflammatory and autoimmune disorders. BM-MSCs possess similar properties, although some reports indicate that AD-MSCs may exert more pronounced anti-inflammatory effects. Fibroblasts do not exhibit such extensive immunoregulatory capabilities. From a safety perspective, both AD-MSCs and BM-MSCs are generally considered relatively safe for clinical applications. The risk of malignant transformation remains low, particularly when cells are cultured for limited periods under stringent quality-control conditions. Dermal fibroblasts are also well-characterized cells that have been used for many years in regenerative medicine and tissue engineering, supporting their favorable safety profile. Nevertheless, their therapeutic potential is generally lower than that of mesenchymal stem cells (Horan et al., 2005; Hass et al., 2011; Driskell and Watt, 2015).

## Clinical application

Wound healing and tissue reconstruction remain major clinical challenges, especially in patients with complex defects resulting from trauma, chronic wounds, or oncological treatments. Cancer patients undergoing surgical resection, radiotherapy, or chemotherapy often experience impaired wound healing, fibrosis, and tissue volume loss, which significantly impact both function and quality of life (Ho et al., 2017). Traditional reconstructive approaches, such as skin grafts or flap surgery, may not always be feasible or sufficient, particularly in irradiated or poorly vascularized tissues (Kondej et al., 2024). This has prompted growing interest in regenerative strategies using autologous adipose tissue and AD-MSCs to enhance tissue repair and modulate the wound microenvironment (Deptuła et al., 2021; Zuk et al., 2001).

Clinically, autologous fat grafting is routinely used in post-oncologic reconstruction, for example, in breast surgery after mastectomy, or in the treatment of radiation-induced skin damage. Beyond volume restoration, fat grafts have been shown to promote angiogenesis, reduce fibrosis, and improve local tissue quality - effects that are largely attributed to the regenerative properties of the stromal vascular fraction (Zuk et al., 2001). A summary of selected clinical outcomes reported for adipose tissue-derived therapies, including graft survival, angiogenesis, and tissue regeneration parameters, is presented in Table 2.

**TABLE 2:** Summary of the most important clinical results obtained after the use of adipose tissue-derived mesenchymal stem cells (AD-MSCs) in regenerative medicine.

Main evaluation indicators	Type of intervention	Clinical outcomes
Graft volume retention, graft survival	Fat grafting enriched with AD-MSCs or SVF	Volume retention and graft survival were higher than with standard fat grafting, with multiple studies reporting 60%–80% volume retention after 3–6 months (Yoshimura et al., 2008)
Skin quality, tissue remodeling	AD-MSCs + fat grafting	Improving collagen organization, skin elasticity and soft tissue quality (Garcia-Arranz et al., 2020)
Vessel density, VEGF expression, neovascularization	AD-MSCs	Increased number of newly formed blood vessels and higher expression of angiogenic factors, especially VEGF (Rehman et al., 2004)
Healing rate, vascularity	AD-MSCs locally or in biomaterials	Acceleration of healing and improvement of vascularization of regenerating tissues (Kim et al., 2007)
Complications, tumor recurrence, fat necrosis	AD-MSCs/SVF	There was no evidence of an increased risk of infection, cysts, fat necrosis, or tumor recurrence in follow-up extending over several years (De Boniface et al., 2018)

Bertolini et al. (2012) From a technical standpoint, there are several methods for delivering adipose tissue and AD-MSCs to the wound site:

- Direct fat grafting, using minimally manipulated lipoaspirate, offers structural support and regenerative benefit, but is limited by unpredictable resorption and poor graft survival in ischemic tissue.
- Isolated stromal vascular fraction (SVF) can be delivered via injection, often mixed with platelet-rich plasma (PRP) or other carriers, and is rich in regenerating cells and cytokines.

The clinical application of adipose tissue and adipose-derived products begins with the harvesting of lipoaspirate, typically performed through manual or suction-assisted liposuction under local or general anesthesia. Common donor sites include the abdomen, flanks, thighs, and buttocks-areas with abundant subcutaneous fat and low morbidity.

In younger patients, abdominal adipose tissue has been shown to exhibit a lower rate of apoptosis and a greater proliferative capacity than that of older individuals (Tan et al., 2016; Fang et al., 2021). Furthermore, lipoaspirated adipose tissue appears to yield fewer stromal cells than excised adipose tissue, likely due to mechanical trauma during harvesting (Vester-Glowinski et al., 2022). Consequently, gentle low-pressure liposuction remains the gold standard for obtaining high-quality lipoaspirate. For small-volume harvests (<100 mL), manual aspiration using a 10-mL Luer-Lok syringe and a blunt cannula with a diameter of at least 2 mm is commonly employed (Fang et al., 2021). The Coleman technique has also served as the standard method of harvesting fat in most studies. However, for large-volume procedures, such as breast reconstruction, low-pressure techniques including water-assisted liposuction (WAL) are preferred because they minimize shear stress on adipocytes. This technique combines high-pressure infiltration of tumescent solution with simultaneous low-pressure suction, allowing the graft to be continuously flushed and filtered while preserving adipocyte integrity (Kølle et al., 2020; Peltoniemi et al., 2013). WAL has been reported to be significantly faster, reducing operative time by approximately 90–150 min, while also lowering costs and potentially reducing the risk of contamination (Peltoniemi et al., 2013).

Most studies favor general anesthesia over local anesthetic infiltration because of the potential cytotoxic effects of lidocaine on adipose-derived cells and its possible impact on cell viability (Peltoniemi et al., 2013). Following aspiration, the lipoaspirate must be processed to concentrate the regenerative cell population. Since the concentration of AD-MSCs in lipoaspirate is approximately half that found in excised adipose tissue, harvesting a volume up to four times greater than the intended graft volume may be necessary to achieve a twofold increase in stem cell numbers (Kølle et al., 2013). Therefore, active enrichment strategies are often employed to increase stem cell yield by 3%–30% (Kølle et al., 2013). Such enrichment may be achieved through enzymatic digestion or mechanical processing, including centrifugation, performed either manually or using automated systems such as Celution (Peltoniemi et al., 2013).

Importantly, no significant differences in SVF cell viability have been observed between conventional lipoaspirate and mechanically emulsified adipose tissue (nanofat) (Sesé et al., 2019). Consequently, nanofat preserves a substantial number of SVF cells following mechanical disaggregation and may provide the regenerative benefits of lipoaspirate while avoiding the cost and time associated with enzymatic processing. Prior to nanofat application, wounds are typically prepared by surgical debridement. In studies investigating scar treatment, emulsified adipose tissue is injected intradermally and directly into scar tissue using sharp needles until blanching of the treated area is observed (Bhooshan et al., 2018; Uyulmaz et al., 2018; Huang et al., 2021; Rageh et al., 2021; Limido et al., 2024). In facial rejuvenation procedures, nanofat is administered both intradermally and subcutaneously (Xu et al., 2018). For wound healing applications and the treatment of alopecia, nanofat has been injected into the wound margins and wound bed or into the scalp, respectively, within the subcutaneous plane (Chen et al., 2019; Li et al., 2020).

At present, no consensus guidelines define the optimal volume of nanofat to be injected. Thamm *et al.* reported transplanting approximately 0.25 mL of nanofat per square centimeter of wound surface area into both the wound bed and surrounding margins (Thamm et al., 2023). Similarly, there is no standardized protocol regarding grafting technique or treatment frequency. Several studies have reported that patients who failed to demonstrate clinical improvement after the initial procedure required two or three additional nanofat grafting sessions to achieve satisfactory outcomes (Bhooshan et al., 2018; Uyulmaz et al., 2018; Xu et al., 2018).

Several technical variables—such as cannula diameter, suction pressure, centrifugation speed, and time—can influence cell viability, yield, and regenerative capacity of the harvested tissue (Con et al., 2016). Studies have shown that gentle handling, low negative pressure, and standardized protocols are essential for maintaining the biological integrity of the graft and maximizing therapeutic efficacy. When autologous fat is used as a source of regenerative cells, the time between harvesting and application should be minimized, and sterility must be ensured throughout to comply with Good Clinical Practice (GCP) and, where applicable, Good Manufacturing Practice (GMP) standards (Sensebé et al., 2013).

However, there are still challenges in the application of adipose tissue-derived therapies in wound healing, such as variability in cell quality, quantity, and viability, as well as differences between individual donors. Additionally, potential risks include infection, aberrant cell proliferation, and tumorigenesis, as well as regulatory issues and the need for larger-scale randomized controlled clinical trials (Hao et al., 2023). To assess the safety and efficacy of AD-MSCs in promoting chronic wound healing, larger-scale randomized controlled trials are still needed (Hao et al., 2023). Tissue regeneration is a complex process, and most of the signaling pathways and mechanisms with use of AD-MSCs need to be further explored (Dong et al., 2023).

### Clinical safety and complications

Complications following autologous fat grafting can be broadly classified as minor or major. Minor complications include erythema, mild edema, prolonged ecchymosis, hematoma, localized pain at the incision site, and oil cyst formation. Major complications include infection, fat graft necrosis, inflammatory infiltrates, abscess formation, fistula development, calcifications, skin necrosis, wound deterioration, fibrosis, severe edema, pain extending beyond the injection site, cellulitis, and fat embolism.

#### Donor-site complications

A review of 416 cases of autologous fat grafting conducted by Homer *et al.* reported an overall complication rate of 5.5%, with donor-site contour irregularities (1.2%) and induration (1.2%) representing the most common adverse events (Homer et al., 2022). Prolonged erythema (0.9%) and infection (0.5%) were reported less frequently. Interestingly, a higher incidence of donor-site complications was associated with a lower body mass index (BMI). Maamari *et al.* additionally described cases of post-inflammatory hyperpigmentation following fat harvesting (Maamari et al., 2019).

#### Recipient-site complications

Fat necrosis is one of the most frequently reported complications of autologous fat grafting (Kølle et al., 2013). The survival of transplanted adipose tissue depends on adequate vascularization and oxygen diffusion from the surrounding recipient tissue. Necrosis may occur when excessive volumes of fat are injected relative to the available recipient surface area, resulting in insufficient perfusion of the central portion of the graft (Cook et al., 2004). In addition, mechanical injury to adipocytes during harvesting, processing, or injection may further compromise graft viability and contribute to fat necrosis. Large areas of fat necrosis (>10 mm) may subsequently develop into oil cysts. The inflammatory response associated with these cysts can lead to calcification. Oil cysts occur more frequently after the injection of large fat volumes into areas such as the breasts or buttocks. Granuloma formation may also occur as a result of a foreign-body inflammatory reaction to contaminants within the graft material (Vester-Glowinski et al., 2022; Yoshimura and Coleman, 2015). In most cases, oil cysts and granulomas can be managed conservatively or by aspiration and drainage, whereas surgical excision is only rarely required. Infection is considered an uncommon complication of autologous fat grafting, with reported incidence rates ranging from 0.10% to 0.54% (Krastev et al., 2018; Evans et al., 2020). However, inadvertent intravascular injection of fat represents one of the most serious complications associated with the procedure. Fat embolism may result in local soft-tissue necrosis and can lead to devastating systemic consequences, including visual loss, ischemic stroke, pulmonary embolism, and death.

### Oncological safety and malignancy potential

Oncological patients present unique challenges for regenerative therapies. Chemotherapy and radiotherapy may reduce the viability or regenerative potential of patient-derived cells, while also altering the wound microenvironment. Studies have shown that AD-MSCs isolated from cancer patients or from irradiated tissues can still exhibit regenerative capacity, but their behavior may differ from cells obtained from healthy donors. Moreover, the oncological safety of using AD-MSCs remains an area of ongoing research, particularly in contexts where residual tumor cells might respond to pro-angiogenic signals. While current evidence does not suggest increased cancer recurrence risk with fat grafting in breast cancer patients, cautious patient selection and long-term follow-up are essential (Bertolini et al., 2012).

The potential risk of tumor induction, particularly in patients with a history of cancer, remains a subject of ongoing debate (Peltoniemi et al., 2013). However, the currently available clinical evidence does not indicate an increased oncological risk within the reported follow-up periods. A pooled analysis of four studies demonstrated that autologous fat grafting was not associated with a significant increase in locoregional recurrence rates (Strong et al., 2024; Lo Torto et al., 2024). Despite these reassuring findings, the identification of patient groups that may be at increased oncological risk remains an important unresolved issue. The available evidence is less robust for biologically aggressive malignancies, including triple-negative breast cancer (TNBC), HER2-positive tumors, and high-grade breast cancers. Most published studies were not sufficiently powered to perform reliable subgroup analyses in these populations. Consequently, concerns regarding these high-risk groups are based primarily on clinical caution rather than strong prospective evidence.

Attention has been directed toward adipose-derived regenerative therapies involving SVF or AD-MSCs enrichment. To date, clinical studies have not demonstrated an increased rate of cancer recurrence associated with these approaches. Nevertheless, investigators consistently emphasize the need for continued surveillance and cautious interpretation of the available data (Peltoniemi et al., 2013; Prieto González and Birbrair, 2019; Calabrese et al., 2018; Valente et al., 2024).

Calabrese *et al.* reported that the use of concentrated SVF was not an independent risk factor for either local recurrence or distant metastasis in patients who had undergone treatment for breast cancer (Calabrese et al., 2018). Interestingly, the control group exhibited a higher incidence of systemic recurrence, including bone and lung metastases, than the SVF-enriched group. However, these findings should be interpreted with caution due to limitations in follow-up duration.

Indeed, one of the major limitations of the current literature is the relatively short follow-up period. Across most studies, the mean follow-up does not exceed 5 years, which is insufficient to establish a definitive oncological safety profile. As breast cancer recurrence may occur many years after primary treatment, often between five and 10 years after diagnosis, long-term surveillance remains essential. Future studies should therefore prioritize extended follow-up periods to determine whether adipose tissue-derived regenerative therapies maintain their oncological safety over a full decade following reconstruction.

## Perspectives and new trends in regeneration

### Acellular adipose matrix as alternative reconstructive method

Recently, an alternative reconstructive method has emerged that remains in the preclinical phase: using acellular adipose matrix (AAM) - the extracellular matrix isolated from adipose tissue) (Kim et al., 2024). AAM acts as a scaffold for stem cell proliferation, adipogenic differentiation, and angiogenesis (Kim et al., 2024; Jin et al., 2025). Initial comparison between conventional fat grafting and AAM showed similar volumetric and regenerative efficacy in soft tissue reconstruction (Kim et al., 2024). AAM shows gradual degradation and integration with surrounding tissues, provides adequate space for cell growth, and promotes proliferation, migration, and differentiation of preadipocytes within it (Yang et al., 2024). Initial results showed that a combination of AD-MSCs and AAM using intraoperative bioprinting (IOB) in reconstruction of full-thickness skin defects is promising (Kang et al., 2011). Implantation into healthy volunteers (abdominal tissue) showed good tolerance and increasing cellular infiltration and immune populations, suggesting ongoing tissue remodeling and potential for long-term tissue replacement (Anderson et al., 2022).

### AD-MSC-derived exosomes in wound healing

Exosomes are small vesicles released by cells that contain various bioactive molecules, including proteins, lipids, and RNA (Kalluri and LeBleu, 2020). These vesicles play a critical role in intercellular communication and have the potential to influence the behavior of neighboring cells (Vučemilović, 2024). In the context of wound healing, exosomes derived from AD-MSCs have been shown to enhance tissue repair and regeneration by modulating inflammation, stimulating the formation of new blood vessels, and promoting cell proliferation and migration (Ma et al., 2020).

Accumulating evidence indicates that many of the previously described immunomodulatory effects of AD-MSCs are in fact mediated by their secreted exosomes–nano-vessels responsible for intercellular communication (Zheng et al., 2021).

Recently, there has been increasing research on the properties and use of AD-MSC-derived exosomes (AD-MSC-Exos). Conditioned medium from AD-MSCs contains multiple factors that increase the production of type I and III collagen and fibronectin (Malekzadeh et al., 2023). AD-MSC-Exos can also accelerate the wound healing process by promoting angiogenesis (VEGFA is described as a main molecule secreted by AD-MSC-Exos that promotes neovascularization) (Zeng et al., 2022; Dong et al., 2023). AD-MSC-Exos promote fibroblast migration, proliferation, and collagen synthesis—processes essential for wound healing (Ferreira and Gomes, 2018). AD-MSCs are effective immune modulators in inflammatory environments, regulating mechanisms related to cell differentiation, proliferation, and migration (Zeng et al., 2022). Through exosome-mediated signaling, they upregulate genes involved in various functions, including skin barrier formation, immune regulation, cell proliferation, and epidermal regeneration^1^. Importantly, the term “exosomes” should be applied in accordance with minimal experimental and reporting standards, such as those proposed by the International Society for Extracellular Vesicles in the MISEV guidelines. These guidelines recommend rigorous characterization of extracellular vesicles, including:

- assessment of particle size distribution and morphology (e.g., nanoparticle tracking analysis, transmission electron microscopy).
- detection of positive protein markers enriched in EVs (e.g., CD9, CD63, CD81, TSG101) together with negative markers to exclude contaminants (e.g., calnexin).
- transparent reporting of isolation and purification methods.

Furthermore functional studies should include appropriate controls to distinguish vesicle-specific effects from co-isolated soluble factors. When these criteria are not fully satisfied, the use of the broader term “extracellular vehicles (EVs)” is recommended instead of “exosomes”. Adherence to these standards is essential for reproducibility and comparability across studies (Witwer et al., 2021; Théry et al., 2018).

AD-MSC-Exos contain many cytokines (e.g., VEGFA, FGF, IGF, and PDGF), which may promote skin wound healing by supporting collagen synthesis, fibroblast proliferation and migration, vascular endothelial cell proliferation, and angiogenesis (Cai et al., 2020).

Beyond their role as natural mediators of intercellular communication, AD-MSC-derived exosomes are increasingly being explored as bioengineered therapeutic carriers. Recent studies have demonstrated the possibility of modifying exosomal cargo, including microRNAs, proteins, and growth factors, to enhance specific regenerative outcomes. This creates opportunities for the development of personalized and targeted regenerative therapies that extend beyond the capabilities of conventional stem cell transplantation. Such approaches represent one of the most rapidly evolving areas of regenerative medicine and tissue engineering (Czerwiec et al., 2023).

Recent studies (2024) have introduced bioengineering strategies to modify exosomes to improve their stability, targeting efficiency, and therapeutic potential. To prolong exosome retention and increase their local concentration within the wound bed, the incorporation of exosomes into biomaterial-based delivery systems has emerged as a promising approach.

To further enhance the effectiveness of exosome-based therapies for wound healing, researchers have developed various engineering strategies aimed at improving exosome loading capacity, controlled release, and biological activity. Currently, these approaches are mainly divided into two categories: direct exosome engineering and stem cell-based exosome engineering. These strategies provide different advantages in terms of cargo delivery efficiency, stability, and therapeutic performance of exosome-based treatments (Sun et al., 2024).

The clinical relevance of these engineering approaches is particularly evident in chronic wounds, where impaired tissue regeneration and vascularization significantly limit the healing process. Treatment-resistant diabetic wounds remain a major clinical challenge due to impaired angiogenesis and persistent inflammation. He *et al.* isolated exosomes derived from AD-MSCs and evaluated their effects in a human vascular endothelial cell model (HUVECs) exposed to high glucose conditions. The authors demonstrated that AD-MSC-derived exosomes improved cell migration and promoted the formation of new vascular structures, which are essential processes for tissue regeneration. Furthermore, the study identified a specific molecular mechanism underlying the therapeutic effects of exosomes through the regulation of the TRIM32/STING signalling axis. AD-MSC-derived exosomes increased TRIM32 protein expression, resulting in reduced STING activity through its modification and degradation. Consequently, the chronic inflammatory response was attenuated, and endothelial cells regained their ability to migrate and form new blood vessels. These findings highlight the potential of AD-MSC-derived exosomes as a promising cell-free therapeutic approach for difficult-to-heal diabetic wounds. However, further studies using animal models and clinical trials are required to confirm their safety, efficacy, and translational potential (He et al., 2024).

The translation of exosome-based therapies into clinical practice requires the development of standardized isolation and characterization protocols, scalable manufacturing strategies, and rigorous safety assessments to prevent unintended immunological or pro-tumorigenic effects (Ferreira and Gomes, 2018).

### Bio-printing

The use of AD-MSCs in 3D printing on a variety of biomaterials, including natural scaffolds made of collagen, fibrin, gelatin, fibronectin, laminin, alginate, and hyaluronic acid, has become popular for biocompatible dressings for hard-to-heal wounds and regeneration of the body’s supporting structures. Owing to their multilineage differentiation and self-renewal potential, 3D printing with AD-MSCs can achieve high cell survival rates of up to 97%. Getova *et al.* justified the use of an ECM-based gel from adipose tissue lipoaspiration as a carrier for immortalized AD-MSCs, which behaved like normal AD-MSCs. The ECM-based 3D ink printing system also promotes the cultivation of fibroblasts that secrete signaling molecules, like those in the body (Sung et al., 2013), which may be important in the treatment of skin defects. The advantages of such a method are the surgical availability of adipose tissue without unwanted side effects, the large volume of material, as well as its use in autologous and allogeneic tissue defects (Getova et al., 2019).

Cryopreservation of fat microparticles after lipoaspiration, followed by printing with collagen, yielded metabolically active cells expressing both pro-inflammatory and anti-inflammatory cytokines for up to 10 days *in vitro*, along with neovascularization—offering an effective approach for producing viable microfat grafts to treat hard-to-heal skin ulcers (Schmitt et al., 2021). Studies of human lipoaspirate 3D printing with alginate, where the lipoaspirate with AD-MSCs, pericytes, EPCs (endothelial progenitor cells) and ECS, proved long-term survival (150 days) of the cells, and the tissue contained vasculature and mature adipocytes. After transplantation into mice, intact human vascular structures were noted inside the construct, in which erythrocytes were found, while the outer layer of vessels was derived from mouse cells (Säljö et al., 2022).

### Epigenetic aspect

An essential component of epidermal homeostasis, and thus wound healing, involves epigenetic regulatory mechanisms. Epigenetic changes regulate biological processes from conception to death. Over the last few years, research has indicated that epigenetic regulatory mechanisms are vital in regulating skin regeneration and implementing repair gene expression programs in the skin epithelium and mesenchyme. Epigenetic regulators are intrinsically involved in skin repair processes and can dynamically regulate keratinocyte proliferation, differentiation, and migration, as well as influence skin regeneration and neoangiogenesis. This is achieved through a series of complex regulatory mechanisms that can both stimulate and inhibit gene activation to transiently alter cell phenotype and behavior and interact with growth factor activity (Lewis et al., 2014).

AD-MSCs co-cultured with human dermal fibroblasts (HDF) were found to express significantly higher levels of miR-29b and miR-21 and have higher proliferation and migration rates than AD-MSCs cultured without HDF. Additionally, increased expression of type I collagen alpha one chain (COL1A1), type III collagen alpha one chain (COL3A1), alpha-smooth muscle actin (α-SMA), VEGF and phosphoinositide 3-kinase (PI3K detected) was observed), p-Akt and reduced expression of phosphatase and tensin homolog (PTEN) and matrix metalloproteinase (MMP)-1. This suggests extracellular remodeling and fibroblast activation and proliferation, and the remodeling of the extracellular matrix (ECM). Thus, AD-MSC-mediated microRNA increase is essential for tissue repair and has therapeutic potential in skin wound healing (Liu et al., 2021).

## Conclusion

Wound healing and tissue reconstruction, particularly in patients with traumatic injuries, chronic wounds, or following cancer treatment, pose significant clinical challenges, and traditional methods are not always effective. Adipose tissue and mesenchymal cells derived from it have considerable regenerative potential and are used clinically, including in autologous fat grafting. Autologous fat grafts improve not only tissue volume but also skin quality and angiogenesis, and reduce fibrosis, effects that are mainly attributed to the action of the SVF.

The effectiveness of these procedures depends on appropriate harvesting and processing techniques, as well as adherence to quality standards. Cell survival and therapeutic efficacy are influenced by cannula diameter, vacuum strength, centrifugation parameters, and the time between collection and administration. Gentle techniques, such as the Coleman technique, are essential for increasing graft survival.

Although AD-MSCs demonstrate promising properties in wound healing, their use remains experimental due to donor heterogeneity, limited cell viability, and insufficient data regarding safety and underlying mechanisms of action. Chemotherapy and radiotherapy can reduce the regenerative potential of these cells. AD-MSCs may retain functionality; however, their behaviour differs from that of cells collected from healthy donors.

While clinical trials are ongoing to evaluate their efficacy, the high costs of advanced cell-based therapies mean that traditional approaches, such as lipotransplantation, are more commonly chosen in clinical practice.
